# Durability Study on High-Performance Fiber-Reinforced Mortar under Simulated Wastewater Pipeline Environment

**DOI:** 10.3390/ma14143781

**Published:** 2021-07-06

**Authors:** Tianyu Wang, Yahong Zhao, Baosong Ma, Cong Zeng

**Affiliations:** 1 Department of Engineering, China University of Geosciences-Wuhan, Wuhan 430074, China; cugwty@cug.edu.cn (T.W.); yhchaos@163.com (Y.Z.); 2 School of Civil Engineering, Sun Yat-Sen University, Zhuhai 519082, China; mabaos@mail.sysu.edu.cn

**Keywords:** durability, high-performance concrete/mortar, wastewater pipeline environments, corrosion

## Abstract

The acid–alkaline-inducd corrosive environments inside wastewater concrete pipelines cause concrete structural deterioration and substantial economic losses all over the world. High-performance concrete/mortar (HPC) was designed to have better resistance to corrosive environments, with enhanced service life. However, the durability of HPC in wastewater pipeline environments has rarely been studied. A high-performance mortar mixture (M) reinforced by supplemental materials (including fly ash and silica fume) and polyvinyl alcohol (PVA) fibers, together with a mortar mixture (P) consisting of cement, sand and water with similar mechanical performance, were both designed and exposed to simulated wastewater pipeline environments. The visual appearance, dimensional variation, mass loss, mechanical properties, permeable pore volume, and microstructure of the specimens were measured during the corrosion cycles. More severe deterioration was observed when the alkaline environment was introduced into the corrosion cycles. Test results showed that the M specimens had less permeable pore volume, better dimensional stability, and denser microstructure than the P specimens under acid–alkaline-induced corrosive environments. The mass-loss rates of the M specimens were 66.1–77.2% of the P specimens after 12 corrosion cycles. The compressive strength of the M specimens was 25.5–37.3% higher than the P specimens after 12 cycles under corrosive environments. Hence, the high-performance mortar examined in this study was considered superior to traditional cementitious materials for wastewater pipeline construction and rehabilitation.

## 1. Introduction

Expanding urbanization spikes the increasing development of underground space [1]. For example, the Singapore government has listed the planning and construction of underground space as a strategic development policy [2]. Elsewhere, other cities, such as Zurich, Kuala Lumpur, and Hong Kong, are trying to have a better understanding of underground utilities by drawing a complex map of the underground space [3]. These underground infrastructures, including pipelines, utility tunnels, and other networks, are the lifelines of a city, connecting different districts like blood vessels [4]. Meanwhile, underground pipeline accidents could cause deleterious social events and economic losses. For example, the loss due to a pipeline accident in China can total billions of dollars, and a 6-billion-gallon loss of treated water per day was estimated in the U.S. due to pipeline failure [5,6]. The long-term operation, corrosion environments, poor design, and humankind’s engineering activities may be the causes of deterioration and failure in a pipeline [7]. Especially in the sewer pipeline system, the complex biological, physical, and chemical processes that happen inside the pipeline easily result in structural deterioration within its design lifetime [8]. Studies show that sewer pipeline system rehabilitation work cost over USD 549 million in Germany, USD 120.7 million in the UK per year, and is estimated to be USD 390 billion in the next 20 years in the USA [9,10,11]. In Hamburg, the sewer concrete pipe corrosion rate was more than 6 cm/year in depth [12]. A field study showed that 100 mm-depth corrosion was observed in the sewer system that was built in 2001 in Edmonton, Canada. The rehabilitation cost was about USD 13.9 million in 2015 [13].

Extensive studies have been conducted to facilitate a better understanding and control of the deterioration process of sewer concrete pipeline structures, through a variety of research works [14,15,16]. Some chemical acids were used as the corrosive agents of the concrete material corrosion. The hydrochloric acid was found to be aggressive to the concrete structure and the steel reinforcement [17]. As for sulfuric acid, the expansibility of its corrosion product could cause secondary damage to the matrix [18,19]. Biological acid produced by Thiobacillus Thiooxidans and other microorganisms was used to simulate the corrosive environment in sewer concrete pipes [20,21,22]. Research showed that the corrosion processes of biological acid and chemical acid were nearly the same, except for the complicated preparatory stages of bio-acid corrosion [23]. Hydrochloric acid was chosen as the acid medium in this study. It was also proven that the changes in temperature and humidity that resulted in dry-wet cycles and freeze-thaw cycles could lead to the accelerated corrosion of the concrete [24,25,26]. The dry-wet cycles were more common and obvious in the sewer system, especially around the waterline area, than other environmental indicators [27]. Thus, the dry-wet cycles were included as one of the corrosive environments in this study.

However, it should be noticed that alkaline wastewater was found to be intermittently transported in the wastewater pipe, while usually an alkaline environment was ignored in the previous long-term studies of the sewer concrete corrosion because of the highly alkaline concrete material [28,29]. However, faster deterioration of the sewer concrete pipe was found around the waterline area under biogenic acid corrosion and alkaline flow with a dry-wet cycle [30]. Until now, most attention was only paid to the acidic corrosion environment [31,32,33], which might make the experimental work and numerical model less practical [32]. Therefore, a dry-wet acid-alkaline cycle condition was designed in this study to simulate the corrosive environment around the waterline area of the wastewater pipeline.

The concept of high-performance concrete (HPC) was developed to fulfill the need for concrete structures with high durability and long service life [34]. Supplemental materials (e.g., fly ash, silica fume), fine aggregate, and fibers were generally introduced into this type of cementitious mixture, with good mechanical and durability performances under corrosive environments [35,36]. This kind of cementitious material has been used in the underground infrastructures’ rehabilitation project [37]. A high-performance fiber-reinforced mortar was designed as a research object in this study. Fly ash (FA), silica fume (SF) and PVA fibers were used to enhance the durability of the material under a simulated corrosion wastewater environment [38,39].

This paper aims to study the deterioration process of cementitious material in a new acid-alkaline cycle corrosive environment, and then develop a high-performance fiber-reinforced mortar that has better resistance to the extremely corrosive environment. The visual appearance, dimensional stability, mass loss, compressive behavior, and microstructure of the cementitious materials were studied during the experimental cycles. The high-performance fiber-reinforced mortar designed in this paper may become a good solution to address problems that happened in the wastewater concrete pipe system. The findings of this study provide a technical reference for understanding the concrete corrosion process and guiding future construction and rehabilitation work of wastewater concrete pipe structures.

## 2. Materials and Methods

### 2.1. Materials

Portland cement ((PC), from Fujian Longlin Group Company Ltd., Fujian, China) conforming to GB/T 14684-2011, was used [40]. The fly ash (FA), which contained SiO_2_ (58%), Al_2_O_3_ (30%), Fe_2_O_3_ (4.3%), CaO (1.5%), MgO (2.8%), Na_2_O (3.2%), and the silica fume (SF) which contained SiO_2_ (≥98%), Al_2_O_3_ (≤0.7%), Fe_2_O_3_ (≤0.6%), CaO (≤0.5%), MgO (≤0.2%), both from Wuhan Newreach Materials (Wuhan, China), were used. The PVA fibers (diameter: 40 μm, length: 12 mm) have a 1.6 GPa tensile strength and Young’s modulus of more than 38 GPa, and were sourced from Kuraray (Tokyo, Japan) and are named Kuralon K-II. The volume fraction of PVA fibers was 1.5%. Silica sand ((SS), silicon dioxide content ≥ 99.3%, Hengwang Environmental Protection, Zhengzhou, China) conforming to GB/T 14684-2011 was used [41]. A polycarboxylate superplasticizer from SUNBO (Suzhou, China) with a water-reducing effect of 30% was used.

Two kinds of cementitious specimens were prepared, as shown in Table 1. The M group was designed with PVA fibers, while the P group was designed to have a similar mechanical performance to the M group. The mixture design is listed in Table 1.

### 2.2. Curing Environments

**Figure 1:**
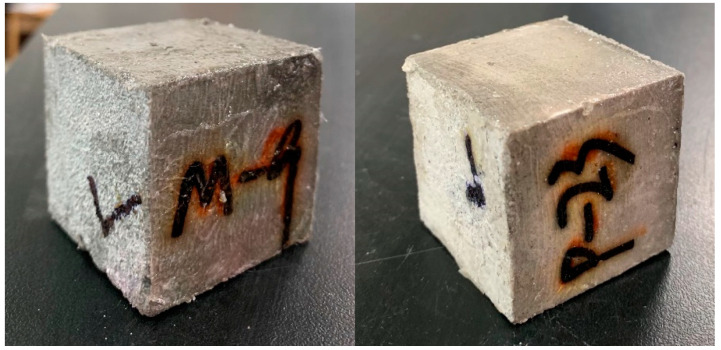
Sealed specimens in the experimental work, the ‘√’ marked surface was the only unsealed surface.

The pH value of the underground pipeline could be reduced by around 1–2 [12], while alkaline wastewater sometimes might have a PH greater than 10 [42]. To simulate the accelerated corrosion environment in a water drainage pipe, 38% hydrochloric acid (HCl), and sodium hydroxide from Xilong Scientific (Guangdong, China) were used to maintain the PH of the acidic environment at around 1 and the alkaline environment at around 13. The corrosion solution would penetrate the matrix through the “√” marked surfaces (Figure 1). Epoxy resin (E51) (Beijing, China) from Beijing Yuhong Waterproof Technology Co. Ltd. was used to seal the other surfaces.

Three different curing environments were designed in Table 2. The specimens were cured in water for the first 7 days, then they were immersed separately in environments I, II and III. In environment I, the specimens were cleaned with water and dried at 20 °C for 24 h after immersion in the acid environment for 4.5 days. Then in the next 4.5 days, the specimens were put into the alkaline environment before the next cycle. The PH values of the acid/alkaline solution were checked with a pH meter (PHS-3C from LeiCi, Hangzhou, China) and maintained every day at around pH 1 and 13, respectively. The M and P specimens were also tested, respectively, after curing in different environments, and comparative analyses were carried out as shown in the following part.

### 2.3. Test Procedures

The visual and mass changes of the specimens were tracked and recorded during the cyclic exposure. The height change perpendicular to the unsealed surface was recorded using a vernier caliper.

The continuous mass-loss rate (R_m_) was determined as follows [43]:

$$R_{m}=\frac{W_{0}-W_{i}}{W_{0}}\times 100\%,\;i=1,2,\ldots ,\;12$$

where W_0_ was the initial weight of the specimens before the cycles, while W_i_ was the weight of the specimens after being exposed to i corrosion cycles. The measurement was carried out by scales with an accuracy of 0.1 mg [44]. The permeable porosity of the specimens was measured per ASTM C642 [45].

**Figure 2:**
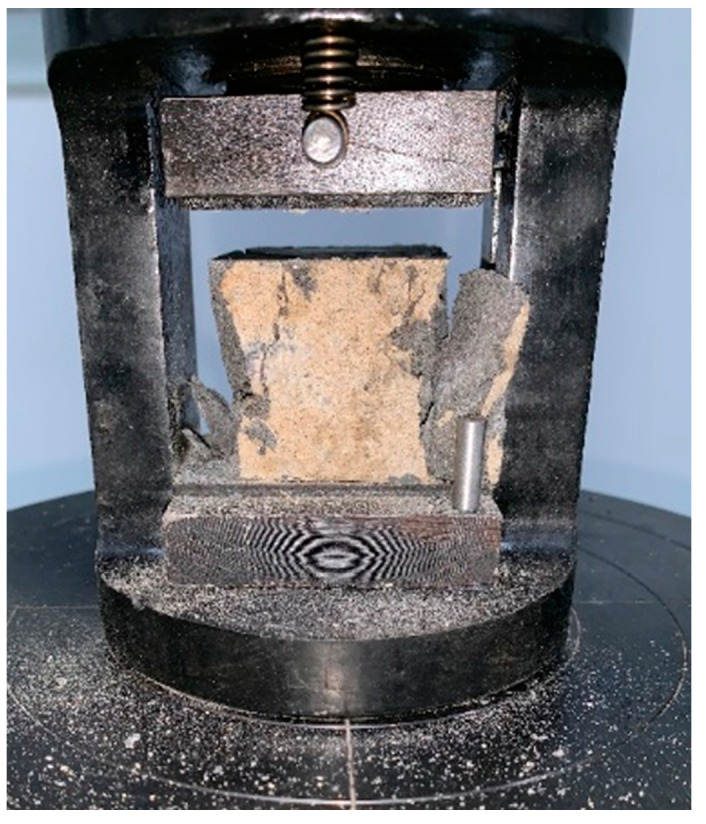
Compression-testing machine.

The cubes (40 × 40 × 40 mm) were tested with a displacement control scheme (SANS YAW4605, MTS System (China) Co., Ltd., Shenzhen, China) to obtain the whole process of compressive behavior (see Figure 2).

The specimens were prepared and Au-sputtered, then measured by JSM-6701F to obtain the FESEM (field emission scanning electron microscope, JEOL Ltd., Tokyo, Japan) images. The crystal structure of the corrosion products was characterized by X-ray diffraction (XRD) using an X’PERT PRO (Philips Corp., Eindhoven, The Netherlands) diffractometer at a glancing angle of 1° with Cu Kα radiation (λ = 0.154 nm) at 2θ ranging from 5 to 60. 

## 3. Results and Discussion

### 3.1. Visual Appearance and Height Loss

**Figure 3:**
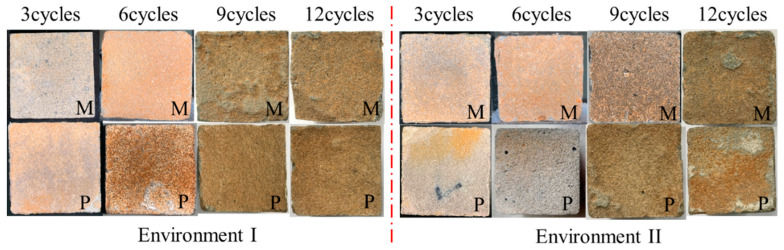
Surface variations of the specimens under corrosion cycles.

The visual appearance changes of the specimens under the corrosion environments are presented in Figure 3. The brownish areas showed up on the surfaces of the specimens because of the iron ion precipitation [46]. The fibers and aggregates were exposed as the corrosion cycles increased. In the later stage, the M specimens under environment I seemed to be more damaged than the specimens under environment II [47]. Both M and P specimens became soft after 12 acid-alkaline cycles, while the surface remained a little bit harder in environment II.

**Figure 4:**
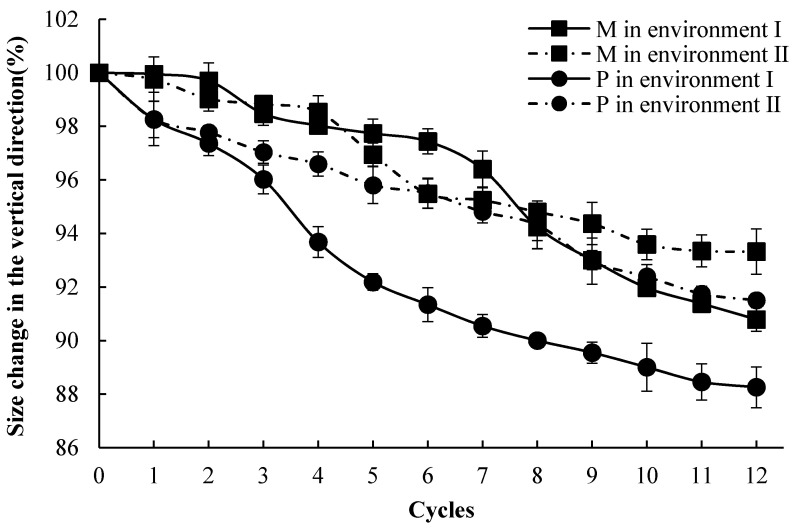
The size change of the specimens under environments I and II.

The size change of the specimens in the vertical direction before and after the corrosion cycles were measured by a vernier caliper (Figure 4). The size of M specimens in the vertical direction became 93.3% and 90.8% of the original ones after 12 cycles of corrosion in environments I and II, respectively. The P specimens lost 8.5% and 11.7% height in the vertical direction under 12 cycles in environments I and II, respectively. The height loss of the M specimens was 79.2% and 79.9% of P specimens, respectively. The height loss of the M and P specimens under environment I was 26.2% and 37.2% more than the specimens in environment II. Worse visual deterioration and height loss of cement specimens were observed under environment I. The results showed that the M specimens showed a better performance regarding visual appearance and dimensional stability under corrosive environments.

### 3.2. Mass Loss Ratio

**Figure 5:**
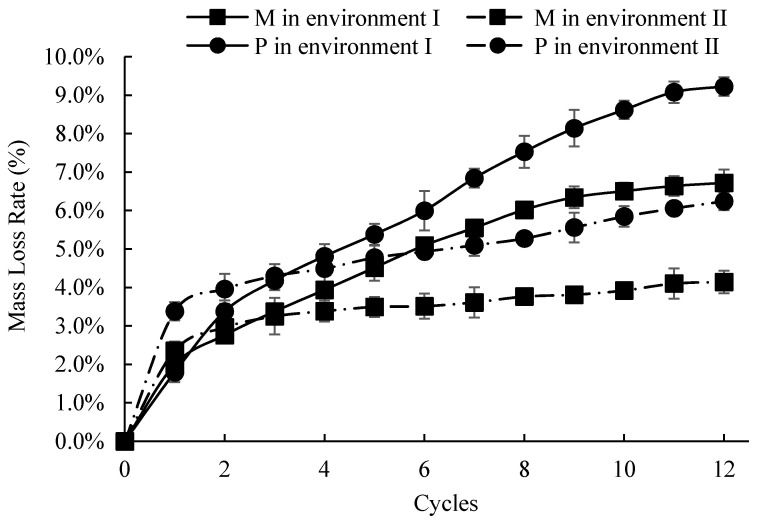
The mass-loss rates of the specimens under 12 cycles of environments I and II.

Except for environment III, a significant mass loss could be observed in both M and P specimens under environments I and II. Figure 5 shows the change of the mass-loss ratio of the specimens during 12 corrosion cycles. After 12 cycles in environment II, the mass-loss rates of the P and M groups were 6.2% and 4.1%, respectively. In addition, the mass-loss rates of P and M under environment I became 9.2% and 7.1%, respectively. The mass-loss rates of the M specimens were 66.1% and 77.2% of the P specimens after 12 corrosion cycles in environments I and II, respectively.

The trends of the mass-loss rates were also slightly different in the two different corrosion environments. In environment II, the curve rises rapidly in the first 1–2 cycles and then tends to be flat in the end. This indicated that the corroded layer slowed down the corrosion rate with the increase of corrosion cycle numbers [48]. Although a smaller rising trend of the mass loss rate curve was observed during the early period of environment I, the rising trend became higher in the later stage. This indicated that the delayed action of the corroded layer might be weakened under the alkaline environment of environment I.

### 3.3. The Permeable Pore Volume

**Figure 6:**
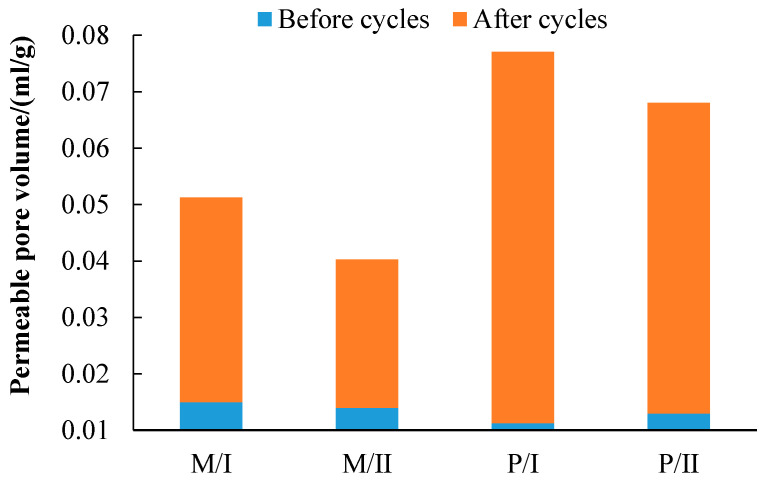
The permeable pore volume of specimens before and after corrosion cycles.

Figure 6 presented the permeable pore volumes for specimens before and after the corrosion cycles. As compared to the P specimens, introducing fiber into the M specimens increased the pore volumes before the corrosion cycles. The pore volumes of all specimens significantly increased after the corrosion cycles. The total pore volume of M and P specimens after 12 cycles in environment I was increased by 37.9% and 19.7% compared with specimens under environment II. The total permeable pore volume of M specimens under 12 cycles in environments I and II were 54.7% and 47.3% of the P specimens, respectively. This indicated that the incorporation of FA and silica fume densified the matrix of M specimens, thus accounting for the enhanced resistance to the corrosive environments [49,50,51,52].

### 3.4. Compressive Strength

**Figure 7:**
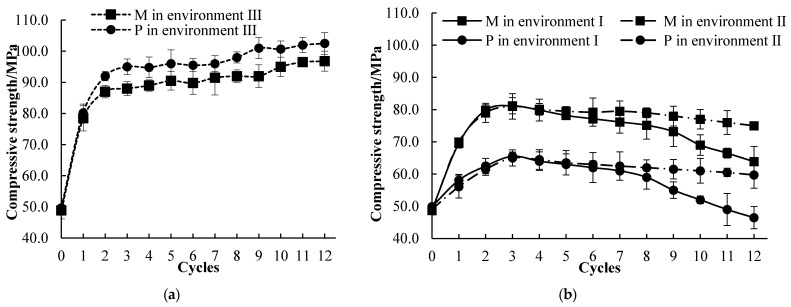
Compressive strength evolution showing (**a**) a generally increasing trend in tap water (environment III), but (**b**) an initial increase followed by a decrease in environments I and II.

Figure 7 showed the compressive strength changes of the M and P specimens immersed in environments I, II and III. The compressive strength of the M and P specimens after 120 d in environment III was increasing from 78.5 and 80.5 MPa to 96.8 and 102.5 MPa, respectively. After 2~3 cycles exposed in environment II, the compressive strength of the M and P specimens increased to a peak and decreased subsequently (Figure 7b). A similar trend was observed in environment III, but the compressive strength decreased more rapidly in the late stage of the corrosion. After 12 cycles, the compressive strength of the P group was only 79.6% and 72.9% of the M group under environments I and II, respectively. The lower mass-loss rates of the M group compared to the P group after the corrosion cycles can be attributed to the supplemental materials (fly ash and silica fume) added to the M group [53]. In the first 2–3 cycles, an increasing trend of compressive strength was observed in environments I and II. This indicated that the hydration process defeated the deterioration effect of the corrosive environments in the early curing period [54]. The 12 cycles in environment I reduced the compressive strength by 34.1% and 54.7%, respectively, for the M and P specimens, and the reduction was 22.52% and 41.7% in environment II, respectively.

The more rapidly decreased trend of the compressive strength of the specimens under environment I indicated that the acid-alkaline cycles could continually cause more damage to the inner cementitious matrix, despite the delaying effect of the corroded layer, especially in the late stages, compared to environment II.

### 3.5. XRD and SEM Analysis

**Figure 8:**
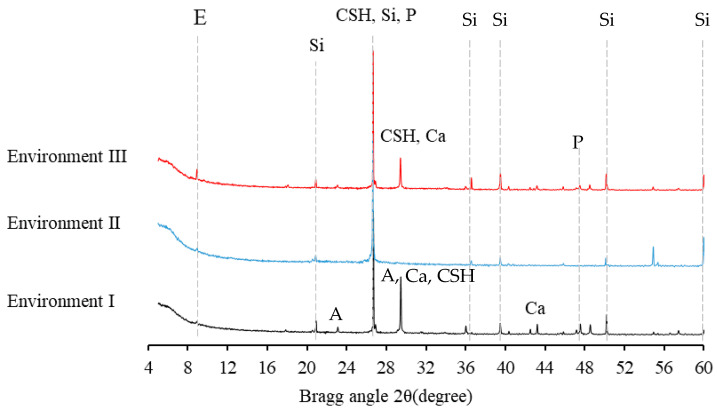
X-ray diffractograms of corrosion products from specimens after immersed in environments I, II and III (E: ettringite, CSH: calcium silicate hydrate, A: albite, Ca: CaCO_3_, P: Ca(OH)_2_, Si: SiO_2_).

**Figure 9:**
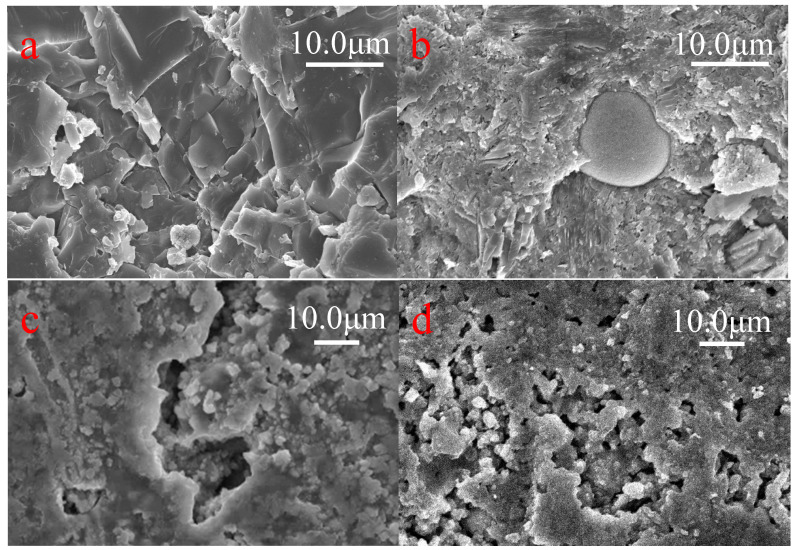

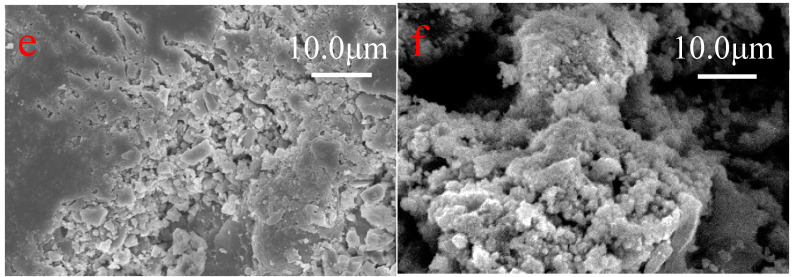
SEM images of M and P specimens under 12 cycles of environments I (**e**,**f**), II (**c**,**d**) and III (**a**,**b**).

The XRD patterns of the specimens cured in different environments are shown in Figure 8. For M and P specimens in tap water, calcium silicate hydrate (C–S–H, 2θ, 29.4°), Ca (OH)_2_(2θ, 26.8° and 47.5°) was identified as a good indicator of cement hydration [55,56]. The compact C-S-H and tabular Ca (OH)_2_ crystal shown in Figure 9a,b also contributed to the good physical and mechanical properties of the cement specimens.

After exposure to environment II (HCl, PH = 1), the calcite (CaCO_3_, 29.4°), ettringite (Ca_6_Al_2_(SO_4_)_3_(OH)12·26H_2_O_8_, 9.0°) and Ca (OH)_2_ were dissolved by the H+ which was indicated by the significant reduction and absent of peaks at 9.0°, 26.8°, 29.4°and 47.5° [57,58]. The silica gel (2θ, 20.9°, 26.8°, 36.6°, 39.5°, 50.2°, 60.0°) was observed in Figure 9c,d with the breaking down of CSH, and the silica gel was identified as the corrosion product accumulating on the surface of the specimens [46]. This phenomenon was conforming to the increase of pore volume and decrease of mass loss and compressive strength.

**Figure 10:**
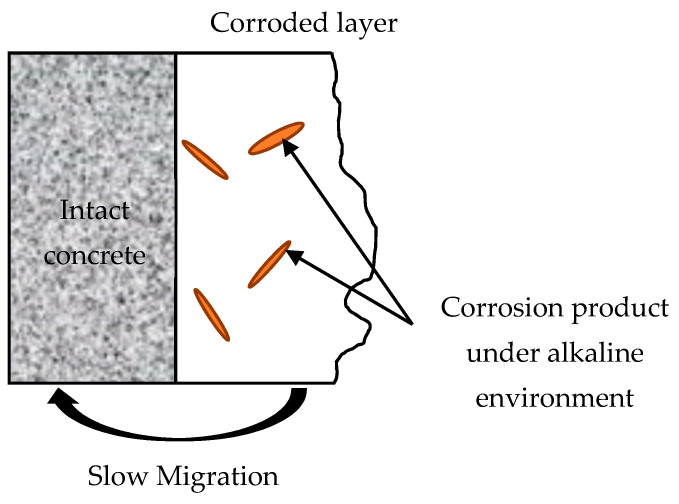
Schematic diagram of the corrosion process under acid/alkaline cycle.

Compared to the XRD pattern in environment II, the albite (Na(AlSi_3_O_8_), 23.2°, 29.5°), Ca (OH)_2_ and more calcite (2θ, 29.5°) were detected in the corrosion product of the specimens under environment I [59]. Unlike the accumulated corrosion products in environment II, the corrosion product under alkaline solution in environment I could create new passages that would accelerate the contact between the acid solution and the intact matrix because of its acid-soluble property (Figure 10). The increasing porosity and non-compact microstructure of the specimens under environments I and II in Figure 9c–f showed the deterioration of the specimens at a microscale, which was consistent with the decreasing trend of mass change and compressive strength in the late curing stage.

## 4. Conclusions

The durability of the high-performance fiber-reinforced mortar was studied under the simulated corrosive environments of the wastewater pipeline. The combined effects of acid, alkaline and wet-dry cycles were investigated. Conclusions can be drawn as follows.

The high-performance fiber-reinforced mortar (M) group had a better visual appearance than the ordinary mortar (P) group after corrosive cycles; the height loss of the M group was less than 80% of the P group after 12 corrosion cycles, which indicated that the M group had better dimensional stability under corrosive environments.

The mass loss ratio and the permeable pore volume of the M group were 66.1–77.2% and 47.3–54.7% of the P group after 12 corrosion cycles, respectively. After 12 corrosion cycles, the compressive strength of the P group was less than 79% of the M group. The better physical and mechanical performance of the M group indicated that the M group had better resistance to the simulated wastewater pipeline environments than the P group.

More serious deterioration was observed on the specimens under environment I (acid-alkaline cycles). The height loss of the M and P groups under environment I was 26.2% and 37.2% more than the height loss under environment II. The mass loss ratio of the M and P groups was 4.1% and 6.2%, respectively, under environment II but increased to 7.1% and 9.2% under environment I. The total pore volume of the M and P specimens after 12 cycles in environment I was increased by 37.9% and 19.7%, respectively, compared with specimens under environment II. The 12 cycles in environment I reduced the compressive strength by 34.1% and 54.7% for M and P specimens, the reduction was 22.52% and 41.7% in environment II, respectively. A greater decreasing trend was observed in the compressive strength curve in the later stage of environment I. The acid-soluble corrosion product (albite) in the alkaline solution could become passages under the following acid environment, which could weaken the delaying effect of the corroded layer on the matrix deterioration rate.

This high-performance fiber-reinforced mortar could become an ideal choice in the wastewater pipeline construction work, according to the experimental results. The experimental work can also provide guidance for wastewater pipeline design and numerical study, based on the high-performance fiber-reinforced mortar under wastewater pipeline environments. Further, more research, such as field tests, needs to be performed to obtain more realistic data.

## Figures and Tables

**Table 1:** Mixture design of M and P specimens (weight ratio).

Mixture ID	M	P
PC (Portland cement)	1	1
FA (fly ash)	0.19	-
SF (silica fume)	0.08	-
SS (silica sand)	1.4	1.4
PVA (% by volume) (polyvinyl alcohol)	1.5	-
Water/cement ratio	0.26	0.26
SP (% by weight) (superplasticizer)	0.12	0.12

**Table 2:** Curing environments of the experimental study.

No.	Curing Environment	The 10-Day Cycle		
		4.5 Days	24 h	4.5 Days
Ⅰ	Acid/Alkaline cycle	PH = 1 acid environment	Dry at 20 °C	PH = 13 alkaline environment
Ⅱ	Acid-only	PH = 1 acid environment		
Ⅲ	Tap water	Tap water (PH around 7)		

## Data Availability

The data presented in this study are openly available in ASCE at American’s infrastructure report card 2021 [6].
